# Availability, acceptability and adoption of decision aids for HIV prevention and contraception for young people: a scoping review protocol

**DOI:** 10.1136/bmjopen-2025-106381

**Published:** 2026-03-04

**Authors:** Itai Kabonga, Oppah Kuguyo, Lindiwe Mancitshana, Kudzai Chidhanguro, Sharon Munhenzva, Fadzai Masiyambiri, Nancy Ruhode, Collin Mangenah, Euphemia Sibanda

**Affiliations:** 1 Centre for Sexual Health and HIV/AIDS Research Zimbabwe, Harare, Zimbabwe; 2 Liverpool School of Tropical Medicine, Liverpool, UK

**Keywords:** Decision Making, HIV & AIDS, PUBLIC HEALTH, Health

## Abstract

**Abstract:**

**Introduction:**

Young people face challenges in accessing information on HIV and sexual and reproductive health services, with corresponding suboptimal uptake. Decision aids can provide information and decisional support to improve young people’s engagement with health interventions. However, they have not been widely implemented among young people. The availability of different choices for HIV and pregnancy prevention means that it is important to implement interventions that facilitate informed choices for these methods. We describe a protocol for a scoping review that aims to explore the availability, acceptability and use of decision aids for HIV prevention and contraception for young people.

**Methods and analysis:**

We will identify relevant studies from the following electronic databases from inception to current date: PubMed, Scopus and Global Health; and grey literature databases, namely medRxiv and Open Access Theses and Dissertations. Eligible studies will report on HIV prevention and/or contraception decision aids and be written in English. Data extraction will be done by two reviewers independently using templates, with discrepancies resolved by consensus. Analysis will be done narratively, and separate for HIV prevention and contraception decision aids. Analysis will also include determination of the suitability of each decision aid for use by young people aged 15–24 years. The Preferred Reporting Items for Systematic Reviews and Meta-Analysis extension for Scoping Reviews will be employed to present results.

**Ethics and dissemination:**

This review does not require ethics approval. The findings from this work will be disseminated through peer-reviewed publications and presentations at local and international conferences.

**Trial registration number:**

This scoping review protocol is registered in Open Science Framework with Project DOI: 10.17605/OSF/IO/46YWG (accessible via: https://doi.org/10.17605/OSF.IO/46YWG).

**STRENGTHS AND LIMITATIONS OF THIS STUDY**

- The study will use a rigorous approach to generate evidence, adhering to the Preferred Reporting Items for Systematic Reviews and Meta-Analysis extension for Scoping Reviews (PRISMA-ScR) guidelines.
- Quality assurance measures such as independent extraction by two reviewers will be implemented to ensure robustness.
- Various electronic databases will be searched to optimise identification of eligible papers.
- A limitation of this study is the potential to omit eligible papers that are not available in English.
- Synthesis will include assessment of suitability of decision aids among young people aged 15–24 years, yet they are a heterogenous group whose characteristics may differ by setting and by age.

## Introduction

 Young people aged 15–24 years have the worst HIV and sexual and reproductive health (SRH) outcomes of all ages globally. Across sub-Saharan Africa, only 65% of young people living with HIV aged 15–24 years know their HIV status, compared with 84% of older adults.^1^ There is suboptimum uptake of condoms and other HIV prevention interventions, including pre-exposure prophylaxis (PrEP), post-exposure prophylaxis (PEP) and voluntary medical male circumcision (VMMC).^2 3^ Every week, 7800 15–24-year-olds are infected with HIV globally,^4^ of whom 25% are African women.^3^ More than 80% of sexually active adolescents in sub-Saharan Africa do not use contraception; millions of young people face unintended pregnancy, unsafe abortions and school drop-out.^5^

Poor engagement in HIV and SRH care and prevention is a huge public health challenge, particularly among young people, for whom the negative health and socioeconomic consequences may persist for decades. Young people face many barriers to the uptake of HIV and SRH services that include fear of disclosure of HIV status/sexual activity, negative attitudes of health workers towards sexual activity in young people and lack of proximity to services.^6 7^ Poor knowledge and poor risk perception are also important: less than 50% of young Africans have comprehensive knowledge of HIV and 48% of those at high risk of infection perceive themselves to be at risk.^6^ In addition, health services fail to provide support to young people in making informed decisions about the range of available options.^2 8^

Decision aids support user-informed choices tailored to their values and preferences and may be applied to provide support on various aspects of care.^9^ For a health intervention with a choice of options, decision aids provide information on each option, including effectiveness, advantages and disadvantages, matched with the specified personal values/preferences. Few decision aids have been evaluated for HIV prevention,^10^ yet the growing number of prevention options (different formulations of PrEP and PEP—tablets, vaginal ring, injectable, formulations with/without contraception, in addition to other prevention options) means that young people need decision support. Decision aids for contraception have been developed and are widely used;^11^ however, there is a need for additional research on how to adopt these contraception decision aids for use in diverse contexts.

We are working with young people and relevant stakeholders to co-develop a peer-driven intervention for supporting uptake of HIV prevention and contraception services among young people. Decision aids for HIV prevention and contraception services will be critical to providing young people with support to make informed choices that are relevant to their circumstances and congruent with their values. To inform the development or adaptation of context-relevant decision aids for contraception and HIV prevention, we aim to conduct a scoping review to explore the availability, acceptability and use of decision aids for HIV prevention and contraception for young people.

## Methodology and methods

### Overview

We will employ a methodological approach of a scoping review to map the extent, nature, range of the literature and potential gaps on the topic.^12^ This scoping review is designed based on the framework described by Arksey and O’Malley^13^ consisting of five stages: (1) identifying the research questions, (2) identifying relevant studies, (3) study selection, (4) charting the data and (5) collating, summarising and reporting the results. These stages are not linear but would be applied iteratively according to the volume and nature of initial findings from reviewed papers. The protocol was registered with Open Science Framework (DOI: 10.17605/OSF.IO/46YWG).^14^ The Preferred Reporting Items for Systematic Reviews and Meta-Analysis (PRISMA) extension for Scoping Reviews guidelines will be used to guide the reporting of the findings. The scoping review process began in October 2024 (drafting of protocol and protocol registration) and is expected to end in July 2026 (dissemination of final findings in a peer-reviewed journal).

### Stage 1: identifying the research question

Research questions for this scoping review were developed primarily through consultation within the research team.^13^ The main research question focuses on determining the availability, acceptability and use/adoption of decision aids for HIV prevention and contraception for young people. The review will be guided by the following specific questions:

- What decision aids are available for HIV prevention services?
- What is the acceptability and adoption of the available decision aids for HIV prevention among young people?
- Are the available decision aids for HIV prevention suitable for low-resource settings?
- What decision aids are available for contraception services?
- What is the acceptability and adoption of the available decision aids for contraception among young people?
- Are the available decision aids for contraception suitable for low-resource settings?

The population, concept and context framework will be used to inform the focus of the scoping review as described below:

Population—young people aged 15–24 years (according to the WHO classification of young people).^15^
Concept—availability, acceptability and adoption of decision aids for HIV prevention and contraception.
Context—global.

### Stage 2: identifying relevant studies

Studies will be identified through searches in electronic databases, reference and citation lists, and grey literature. Search strategies will be developed with the help of a librarian. Electronic databases including PubMed, Scopus and Global Health will be searched. They will be searched using a search strategy that combines subject headings and free-text terms/strings for HIV prevention and contraception decision aids (table 1 and online supplemental appendix 1). Relevant studies will be identified based on the following inclusion and exclusion criteria:

**Table 1:** Search strategy

Search line	Search terms category
1	HIV prevention methods with adjacency search terms
2	HIV prevention methods without adjacency searches
3	Drug/formulation names for products used as HIV PrEP
4	Subject headings for HIV prevention methods (unique to each database)
**5**	**1 OR 2 OR 3 OR 4**
6	Contraception methods
7	Subject headings for contraception methods (unique to each database)
**8**	**6 OR 7**
9	Decision aid with adjacency search terms
10	Decision aid without adjacency searches
11	Subject headings for decision aid (unique to each database)
**12**	**9 OR 10 OR 11**
**13**	**5 OR 8**
**14**	**12 AND 13**

See online supplemental appendix 1 for full search strategy for PubMed to be adapted to all databases.

Bold values indicate where search lines will be combined and how they will be combined using operators OR and AND.

PrEP, pre-exposure prophylaxis.

#### Inclusion criteria

- Papers reporting on specific decision aids for either HIV prevention or contraception.
- All study designs.

#### Exclusion criteria

- Papers that are not in English.

Search terms will initially be tested in PubMed to assess the sensitivity of the search terms before the comprehensive search in all selected databases. The searching of databases will be iterative, and indicator publications will be used to assess whether the search strategy is identifying papers we know to be eligible. If the search does not identify the indicator publications, we will revise the search terms until the indicator publications are picked. We will also conduct backward and forward citation chasing in reference lists of eligible articles.

We will also conduct searches in engines such as Open Access Theses and Dissertations and medRxiv. Across these platforms, we will use a combination of a few key words to identify relevant grey literature that includes organisational reports, research reports, government reports, pre-prints and dissertations/theses.

### Stage 3: selection of eligible papers

All sources identified through the comprehensive search will be downloaded onto EndNote (Clarivate, Philadelphia, USA)^16^ reference manager for sorting and deduplication followed by exporting to Covidence.^17^ Seven reviewers doing single reviewer screening with verification^18^ will screen papers for eligibility in Covidence, based on the article title and abstract, and where relevant the full text, according to inclusion criteria. Each reviewer’s screened papers will be subjected to 10% quality control by independent reviewers to check adherence to eligibility criteria.^18^ The selection process, including reasons for excluding papers, will be summarised in a PRISMA diagram in the final scoping review report.

### Stage 4: charting the data

We will develop a data charting form in Microsoft Excel to abstract data summarised in table 2 from all studies that are included in this scoping review. A pilot data charting exercise will be conducted on 5–10 randomly selected publications, by two independent reviewers to test the data extraction tool for completeness,^19^,^21^ as well as determine the consistency and accuracy of the charting process. Two independent reviewers will extract data from the selected full text articles onto the finalised data charting form. Comparison for accuracy and consistency between the reviewers will be conducted iteratively, with discrepancies resolved through consensus. The team will be open to revising the charting tool during the process of data collection if the need arises and changes made will be documented in the full scoping review report. Authors of full texts may be contacted for clarification during the extraction process, if needed.

**Table 2:** Summary of data to be collected in articles during the data charting process

Category	Details
General characteristics	Study identification (author name, year) Type of article (journal article, thesis, organisational report etc). Period of study. Country of origin.
Study characteristics	Aim of the study. Study design. Targeted population (age, sex). Sampling and sample size.
Study findings	Categorise according to: Who developed decision aid. Focus of decision aid (HIV prevention or contraception). Type of decision aid (client centred, health worker centred, both). Format of decision aid (print, electronic). Detailed description of the decision aid including the year of development, available adaptations of the decision aid, and year of adaptation. To ensure effective comparisons, we will treat adaptations of decision aids that are beyond language translation or format (eg, from print to digital) as independent decision aid tools. Reported suitability/appropriateness, adoption/use and acceptability of decision aid for study population Suitability/appropriateness, adoption/use and acceptability of decision aid for young people—based on findings reported within the manuscript and an assessment tool adapted from *WHO Quality assessment Guidebook: A guide to assessing health services for adolescents.*^22^

This scoping review also aims to determine the suitability and appropriateness of existing HIV prevention and contraception decision aids for use by young people. To assess the suitability/appropriateness of the decision aids for young people, we have developed a tool (online supplemental appendix 2) adapted from the WHO Quality assessment Guidebook: A guide to assessing health services for adolescents.^22^ The tool comprises five domains looking at whether the decision aid is accessible, acceptable, appropriate, effective and equitable.

### Stage 5: collating, summarising and reporting the results

The PRISMA flowchart will be used to visualise the screening process giving an overview of included and excluded studies. The results of the scoping review will be reported quantitatively through numerical analysis (frequency counts) and qualitatively through narrative synthesis. Numerical counts will highlight the number of publications reporting decision aids, counts of each decision aid type, types of studies reported and counts of decision aids that are suitable for young people. Tables and charts will be used to present the numerical results. Narrative synthesis will follow the thematic analysis approach to identify common patterns and ideas in the data on availability, acceptability and adoption of decision aids for HIV prevention and contraception. We will also conduct narrative comparisons across time periods represented in the review.

## Ethics and dissemination

Ethical approval will not be sought for this scoping review as data will be collected from already published academic and grey literature. The results of the scoping review will be disseminated in a peer-reviewed journal and appropriate conference and to stakeholders involved in HIV and SRH in Zimbabwe.

### Patient and public involvement

Patients or the public were not involved in the design, conduct, reporting or dissemination plans of our research.

## Supplementary material

10.1136/bmjopen-2025-106381 online supplemental file 1

10.1136/bmjopen-2025-106381 online supplemental file 2
