# Supplementary material for: Availability, acceptability and adoption of decision aids for HIV prevention and contraception for young people: a scoping review protocol
Source: BMJ Open. 2026 Mar 4;16(3):e106381. doi: 10.1136/bmjopen-2025-106381 (PMC12970088; doi:10.1136/bmjopen-2025-106381)
Supplement: online supplemental file 1 [file bmjopen-16-3-s001.docx]

**Appendix 1: Full search strategy for PubMed to be adapted to Global Health, CINHAL, SCOPUS, and Africa Journals Online databases**

| **Line** | **Search terms** |
| --- | --- |
| 1 | "HIV prevention"[Title/Abstract:~5] OR "male circumcision"[Title/Abstract:~3] OR "circumcised male"[Title/Abstract:~3] OR "circumcised males"[Title/Abstract:~3] OR "long-acting cabotegravir"[Title/Abstract:~2] OR "injectible cabotegravir"[Title/Abstract:~3] OR "cabotegravir injection"[Title/Abstract:~3] OR "cabotegravir injections"[Title/Abstract:~3] OR "long-acting lenacapavir"[Title/Abstract:~2] OR "lenacapavir injection"[Title/Abstract:~3] OR "lenacapavir injections"[Title/Abstract:~3] OR "Dapivirine ring"[Title/Abstract:~3] OR "HIV test"[Title/Abstract:~2] OR "HIV tests"[Title/Abstract:~2] OR "HIV tested"[Title/Abstract:~2] OR "HIV testing"[Title/Abstract:~2] OR "HIV screen"[Title/Abstract:~2] OR "HIV screened"[Title/Abstract:~2] OR "HIV screening"[Title/Abstract:~2] |
| 2 | "pre-exposure prophylaxis"[Title/Abstract] OR "post-exposure prophylaxis"[Title/Abstract] OR "traditional circumcision"[Title/Abstract] OR PrEP[Title/Abstract] OR PEP[Title/Abstract] OR PEPSE[Title/Abstract] OR VMMC[Title/Abstract] OR CAB-LA[Title/Abstract] OR TDF/FTC[Title/Abstract] OR "tenofovir alafenamide"[Title/Abstract] OR F/TAF[Title/Abstract] OR HIVST[Title/Abstract])))) OR (((((((((("HIV TRI-DOT"[Title/Abstract]) OR ("CABENUVA"[Title/Abstract])) OR ("Isentress"[Title/Abstract])) OR ("OraQuick"[Title/Abstract])) OR ("INSTI"[Title/Abstract])) OR ("TRUVADA"[Title/Abstract])) OR ("Apretude"[Title/Abstract])) OR (“Determine HIV-1/2”[Title/Abstract])) OR ("ChemBio"[Title/Abstract])) OR (“Sunlenca”[Title/Abstract])) |
| 3 | (tenofovir alafenamide[Title/Abstract] AND emtricitabine[Title/Abstract]) OR (((("tenofovir disoproxil fumarate"[Title/Abstract]) AND (emtricitabine[Title/Abstract]))))) |
| 4 | "HIV Infections/prevention and control"[Mesh] OR "HIV Infections/transmission"[Mesh] )) OR ( "Acquired Immunodeficiency Syndrome/prevention and control"[Mesh] OR "Acquired Immunodeficiency Syndrome/transmission"[Mesh] )) OR "HIV Seropositivity/transmission"[Mesh]) OR "HIV Testing"[Mesh]) OR "Anti-HIV Agents"[Mesh]) OR "Emtricitabine, Tenofovir Disoproxil Fumarate Drug Combination"[Mesh]) OR "Pre-Exposure Prophylaxis"[Mesh]) OR "Post-Exposure Prophylaxis"[Mesh]) OR "Circumcision, Male"[Mesh]) OR "cabotegravir" [Supplementary Concept]) OR "lenacapavir" [Supplementary Concept]) OR "tenofovir alafenamide" [Supplementary Concept]) OR "Dapivirine" [Supplementary Concept])) |
| **5** | **1 OR 2 OR 3 OR 4**  ((((tenofovir alafenamide[Title/Abstract] AND emtricitabine[Title/Abstract]) OR (((("tenofovir disoproxil fumarate"[Title/Abstract]) AND (emtricitabine[Title/Abstract])))))) OR ("HIV Infections/prevention and control"[Mesh] OR "HIV Infections/transmission"[Mesh] )) OR ( "Acquired Immunodeficiency Syndrome/prevention and control"[Mesh] OR "Acquired Immunodeficiency Syndrome/transmission"[Mesh] )) OR "HIV Seropositivity/transmission"[Mesh]) OR "HIV Testing"[Mesh]) OR "Anti-HIV Agents"[Mesh]) OR "Emtricitabine, Tenofovir Disoproxil Fumarate Drug Combination"[Mesh]) OR "Pre-Exposure Prophylaxis"[Mesh]) OR "Post-Exposure Prophylaxis"[Mesh]) OR "Circumcision, Male"[Mesh]) OR "cabotegravir" [Supplementary Concept]) OR "lenacapavir" [Supplementary Concept]) OR "tenofovir alafenamide" [Supplementary Concept]) OR "Dapivirine" [Supplementary Concept])))) OR ("pre-exposure prophylaxis"[Title/Abstract] OR "post-exposure prophylaxis"[Title/Abstract] OR "traditional circumcision"[Title/Abstract] OR PrEP[Title/Abstract] OR PEP[Title/Abstract] OR PEPSE[Title/Abstract] OR VMMC[Title/Abstract] OR CAB-LA[Title/Abstract] OR TDF/FTC[Title/Abstract] OR "tenofovir alafenamide"[Title/Abstract] OR F/TAF[Title/Abstract] OR HIVST[Title/Abstract])))) OR (((((((((("HIV TRI-DOT"[Title/Abstract]) OR ("CABENUVA"[Title/Abstract])) OR ("Isentress"[Title/Abstract])) OR ("OraQuick"[Title/Abstract])) OR ("INSTI"[Title/Abstract])) OR ("TRUVADA"[Title/Abstract])) OR ("Apretude"[Title/Abstract])) OR (Determine HIV-1/2[Title/Abstract])) OR ("ChemBio"[Title/Abstract])) OR (Sunlenca[Title/Abstract])))) OR ("HIV prevention"[Title/Abstract:~5] OR "male circumcision"[Title/Abstract:~3] OR "circumcised male"[Title/Abstract:~3] OR "circumcised males"[Title/Abstract:~3] OR "long-acting cabotegravir"[Title/Abstract:~2] OR "injectible cabotegravir"[Title/Abstract:~3] OR "cabotegravir injection"[Title/Abstract:~3] OR "cabotegravir injections"[Title/Abstract:~3] OR "long-acting lenacapavir"[Title/Abstract:~2] OR "lenacapavir injection"[Title/Abstract:~3] OR "lenacapavir injections"[Title/Abstract:~3] OR "Dapivirine ring"[Title/Abstract:~3] OR "HIV test"[Title/Abstract:~2] OR "HIV tests"[Title/Abstract:~2] OR "HIV tested"[Title/Abstract:~2] OR "HIV testing"[Title/Abstract:~2] OR "HIV screen"[Title/Abstract:~2] OR "HIV screened"[Title/Abstract:~2] OR "HIV screening"[Title/Abstract:~2]) |
| 6 | contracepti*[Title/Abstract] OR "birth control"[Title/Abstract] OR "fertility control"[Title/Abstract] OR "progest* only"[Title/Abstract] OR "mini pill"[Title/Abstract] OR "combination pill"[Title/Abstract] OR "family planning"[Title/Abstract] OR condom*[Title/Abstract] OR "intrauterine device*"[Title/Abstract] OR IUD[Title/Abstract] OR IUDs[Title/Abstract] OR "vaginal ring*"[Title/Abstract] OR "vaginal sponge*"[Title/Abstract] OR "vaginal shield*"[Title/Abstract] OR "vaginal diaphragm*"[Title/Abstract] OR "cervical cap*"[Title/Abstract] OR Jadelle[Title/Abstract] OR Implanon[Title/Abstract] OR Nexplanon[Title/Abstract] OR "levonorgestrel implant*"[Title/Abstract] OR "etonogestrel implant*"[Title/Abstract] OR NuvaRing[Title/Abstract] OR Annovera[Title/Abstract] OR EluRyng[Title/Abstract] OR LARC[Title/Abstract] OR "depot medroxyprogesterone acetate"[Title/Abstract] OR Depo-Provera[Title/Abstract] OR DMPA[Title/Abstract] OR "Depo-SubQ Provera 104"[Title/Abstract] OR "medroxyprogesterone acetate"[Title/Abstract] OR "Sayana Press"[Title/Abstract] OR "norethisterone enanthate"[Title/Abstract] OR "norethindrone enanthate"[Title/Abstract] OR Levonelle[Title/Abstract] OR "morning after pill*"[Title/Abstract] OR "rhythm method"[Title/Abstract] OR "withdrawal method"[Title/Abstract] OR "coitus interruptus"[Title/Abstract] OR "ovulation method"[Title/Abstract] OR "standard days method"[Title/Abstract] OR "calendar method"[Title/Abstract] OR "lactational amenorrhea method"[Title/Abstract] OR "sexual abstinence"[Title/Abstract] OR "periodic abstinence"[Title/Abstract] OR "male sterilisation*"[Title/Abstract] OR "female sterilisation*"[Title/Abstract] OR "male sterilization*"[Title/Abstract] OR "female sterilization*"[Title/Abstract] OR vasectomy[Title/Abstract] OR "vas ligation*"[Title/Abstract] OR "tubal ligation*") OR “Drospirenone”[Title/Abstract] OR “Azurette”[Title/Abstract] OR ”Microgynon”[Title/Abstract] OR “Aviane”[Title/Abstract] OR “Desogestrel”[Title/Abstract] OR “Lessina”[Title/Abstract] OR “Levora”[Title/Abstract] OR “Loestrin”[Title/Abstract] OR “Lutera”[Title/Abstract] OR “rigevidon” [Title/Abstract] OR “Apri” [Title/Abstract] OR “colique”[Title/Abstract] OR “Cilique”[Title/Abstract] OR “Lo/ovral-28”[Title/Abstract] OR “Ortho-Novum”[Title/Abstract] OR “Portia”[Title/Abstract] OR “Drospirenone/Ethinyl estradiol”[Title/Abstract] OR “Alesse”[Title/Abstract] OR “Levonorgestrel”[Title/Abstract] OR “Natazia”[Title/Abstract] OR “ortho tri-cyclen”[Title/Abstract] OR “nuvaring”[Title/Abstract] OR “annovera”[Title/Abstract] OR “EluRyng”[Title/Abstract] OR “etonogestrel/ ethynyl estradiol”[Title/Abstract] OR “segestrone acetate/ ethinyl estradiol”[Title/Abstract] OR “norlevo”[Title/Abstract] OR “postinor”[Title/Abstract] OR “Noriday”[Title/Abstract] OR “Xulane”[Title/Abstract] OR “Twirla”[Title/Abstract] OR “zafemy”[Title/Abstract] OR “Evra”[Title/Abstract] OR “Ortho Evra”[Title/Abstract] OR “Mirena”[Title/Abstract] OR “Liletta”[Title/Abstract] OR “Kyleena”[Title/Abstract] OR “Skyla”[Title/Abstract] OR “Paragard”[Title/Abstract])) OR “Copper T380A”[Title/Abstract])) OR “LNg 20”[Title/Abstract]) OR “Nova T”[Title/Abstract] OR “spermicide”[Title/Abstract] OR “symptothermal”[Title/Abstract] OR “post ovulation coitus*”[Title/Abstract] OR “periodic abstinence”[Title/Abstract] OR “vaginal douche”[Title/Abstract] OR “option 2”[Title/Abstract] OR “unwanted pregcard”[Title/Abstract] OR “after a”[Title/Abstract] OR “unwanted72”[Title/Abstract])) OR “plan b”[Title/Abstract] OR “Ella one”[Title/Abstract] OR “Ellaone”[Title/Abstract] OR “My choice”[Title/Abstract] OR “take action”[Title/Abstract] OR “after pill”[Title/Abstract] OR “afterpill” [Title/Abstract] OR “herstyle”[Title/Abstract] OR “her style”[Title/Abstract] OR “opcicon onestep”[Title/Abstract] OR “opcicon one step”[Title/Abstract] OR “Plan B”[Title/Abstract] OR “Ulipristal”[Title/Abstract] OR “Next Choice”[Title/Abstract])) OR “New Day”[Title/Abstract] OR “Ortho All-flex diaphragm”[Title/Abstract] OR “Cap diaphragm”[Title/Abstract] OR “Milex”[Title/Abstract] OR “Caya”[Title/Abstract] OR “Postinor”[Title/Abstract] OR “Escapelle”[Title/Abstract] OR “Cerazette”[Title/Abstract] OR “Yasmin”[Title/Abstract] OR “Norgeston”[Title/Abstract] OR “Marvelon”[Title/Abstract] OR “Femilion”[Title/Abstract] OR “Norlutin”[Title/Abstract] OR “Novafem”[Title/Abstract] OR “Mesigyna”[Title/Abstract])) OR “Lunelle”[Title/Abstract] OR “feminena”[Title/Abstract] OR “Cyclofem”[Title/Abstract] OR “Noristerat”[Title/Abstract] OR “NorLevo”[Title/Abstract] |
| 7 | (((((((("Contraception"[Mesh]) OR "Contraceptive Agents"[Mesh]) OR "Family Planning Services"[Mesh]) OR "Contraceptive Devices"[Mesh]) OR "Vasectomy"[Mesh]) OR "Sterilization, Tubal"[Mesh]) OR "Medroxyprogesterone Acetate"[Mesh]) OR "norethindrone enanthate" [Supplementary Concept] |
| **8** | **6 or 7**  ((((((((("Contraception"[Mesh]) OR "Contraceptive Agents"[Mesh]) OR "Family Planning Services"[Mesh]) OR "Contraceptive Devices"[Mesh]) OR "Vasectomy"[Mesh]) OR "Sterilization, Tubal"[Mesh]) OR "Medroxyprogesterone Acetate"[Mesh]) OR "norethindrone enanthate" [Supplementary Concept]) OR (contracepti*[Title/Abstract] OR "birth control"[Title/Abstract] OR "fertility control"[Title/Abstract] OR "progest* only"[Title/Abstract] OR "mini pill"[Title/Abstract] OR "combination pill"[Title/Abstract] OR "family planning"[Title/Abstract] OR condom*[Title/Abstract] OR "intrauterine device*"[Title/Abstract] OR IUD[Title/Abstract] OR IUDs[Title/Abstract] OR "vaginal ring*"[Title/Abstract] OR "vaginal sponge*"[Title/Abstract] OR "vaginal shield*"[Title/Abstract] OR "vaginal diaphragm*"[Title/Abstract] OR "cervical cap*"[Title/Abstract] OR Jadelle[Title/Abstract] OR Implanon[Title/Abstract] OR Nexplanon[Title/Abstract] OR "levonorgestrel implant*"[Title/Abstract] OR "etonogestrel implant*"[Title/Abstract] OR NuvaRing[Title/Abstract] OR Annovera[Title/Abstract] OR EluRyng[Title/Abstract] OR LARC[Title/Abstract] OR "depot medroxyprogesterone acetate"[Title/Abstract] OR Depo-Provera[Title/Abstract] OR DMPA[Title/Abstract] OR "Depo-SubQ Provera 104"[Title/Abstract] OR "medroxyprogesterone acetate"[Title/Abstract] OR "Sayana Press"[Title/Abstract] OR "norethisterone enanthate"[Title/Abstract] OR "norethindrone enanthate"[Title/Abstract] OR Levonelle[Title/Abstract] OR "morning after pill*"[Title/Abstract] OR "rhythm method"[Title/Abstract] OR "withdrawal method"[Title/Abstract] OR "coitus interruptus"[Title/Abstract] OR "ovulation method"[Title/Abstract] OR "standard days method"[Title/Abstract] OR "calendar method"[Title/Abstract] OR "lactational amenorrhea method"[Title/Abstract] OR "sexual abstinence"[Title/Abstract] OR "periodic abstinence"[Title/Abstract] OR "male sterilisation*"[Title/Abstract] OR "female sterilisation*"[Title/Abstract] OR "male sterilization*"[Title/Abstract] OR "female sterilization*"[Title/Abstract] OR vasectomy[Title/Abstract] OR "vas ligation*"[Title/Abstract] OR "tubal ligation*") OR "Drospirenone"[Title/Abstract] OR "Azurette"[Title/Abstract] OR "Microgynon"[Title/Abstract] OR "Aviane"[Title/Abstract] OR "Desogestrel"[Title/Abstract] OR "Lessina"[Title/Abstract] OR "Levora"[Title/Abstract] OR "Loestrin"[Title/Abstract] OR "Lutera"[Title/Abstract] OR "rigevidon" [Title/Abstract] OR "Apri" [Title/Abstract] OR "colique"[Title/Abstract] OR "Cilique"[Title/Abstract] OR "Lo/ovral-28"[Title/Abstract] OR "Ortho-Novum"[Title/Abstract] OR "Portia"[Title/Abstract] OR "Drospirenone/Ethinyl estradiol"[Title/Abstract] OR "Alesse"[Title/Abstract] OR "Levonorgestrel"[Title/Abstract] OR "Natazia"[Title/Abstract] OR "ortho tri-cyclen"[Title/Abstract] OR "nuvaring"[Title/Abstract] OR "annovera"[Title/Abstract] OR "EluRyng"[Title/Abstract] OR "etonogestrel/ ethynyl estradiol"[Title/Abstract] OR "segestrone acetate/ ethinyl estradiol"[Title/Abstract] OR "norlevo"[Title/Abstract] OR "postinor"[Title/Abstract] OR "Noriday"[Title/Abstract] OR "Xulane"[Title/Abstract] OR "Twirla"[Title/Abstract] OR "zafemy"[Title/Abstract] OR "Evra"[Title/Abstract] OR "Ortho Evra"[Title/Abstract] OR "Mirena"[Title/Abstract] OR "Liletta"[Title/Abstract] OR "Kyleena"[Title/Abstract] OR "Skyla"[Title/Abstract] OR "Paragard"[Title/Abstract])) OR "Copper T380A"[Title/Abstract])) OR "LNg 20"[Title/Abstract]) OR "Nova T"[Title/Abstract] OR "spermicide"[Title/Abstract] OR "symptothermal"[Title/Abstract] OR "post ovulation coitus*"[Title/Abstract] OR "periodic abstinence"[Title/Abstract] OR "vaginal douche"[Title/Abstract] OR "option 2"[Title/Abstract] OR "unwanted pregcard"[Title/Abstract] OR "after a"[Title/Abstract] OR "unwanted72"[Title/Abstract])) OR "plan b"[Title/Abstract] OR "Ella one"[Title/Abstract] OR "Ellaone"[Title/Abstract] OR "My choice"[Title/Abstract] OR "take action"[Title/Abstract] OR "after pill"[Title/Abstract] OR "afterpill" [Title/Abstract] OR "herstyle"[Title/Abstract] OR "her style"[Title/Abstract] OR "opcicon onestep"[Title/Abstract] OR "opcicon one step"[Title/Abstract] OR "Plan B"[Title/Abstract] OR "Ulipristal"[Title/Abstract] OR "Next Choice"[Title/Abstract])) OR "New Day"[Title/Abstract] OR "Ortho All-flex diaphragm"[Title/Abstract] OR "Cap diaphragm"[Title/Abstract] OR "Milex"[Title/Abstract] OR "Caya"[Title/Abstract] OR "Postinor"[Title/Abstract] OR "Escapelle"[Title/Abstract] OR "Cerazette"[Title/Abstract] OR "Yasmin"[Title/Abstract] OR "Norgeston"[Title/Abstract] OR "Marvelon"[Title/Abstract] OR "Femilion"[Title/Abstract] OR "Norlutin"[Title/Abstract] OR "Novafem"[Title/Abstract] OR "Mesigyna"[Title/Abstract])) OR "Lunelle"[Title/Abstract] OR "feminena"[Title/Abstract] OR "Cyclofem"[Title/Abstract] OR "Noristerat"[Title/Abstract] OR "NorLevo"[Title/Abstract]) |
| 9 | "decision aid"[Title/Abstract:~4] OR "decision aids"[Title/Abstract:~4] OR "decision tool"[Title/Abstract:~4] OR "decision tools"[Title/Abstract:~4] OR "decision support"[Title/Abstract:~4] OR "decision making"[Title/Abstract:~3] OR "decision instrument"[Title/Abstract:~4] OR "decision instruments" [Title/Abstract:~4] OR "decision technology"[Title/Abstract:~4] OR "decision technologies"[Title/Abstract:~4] OR "decision technique"[Title/Abstract:~4] OR "decision techniques"[Title/Abstract:~4] OR "decision system"[Title/Abstract:~4] OR "decision systems"[Title/Abstract:~4] OR "decision program"[Title/Abstract:~4] OR "decision programs"[Title/Abstract:~4] OR "decision programme"[Title/Abstract:~4] OR "decision programmes"[Title/Abstract:~4] OR "decision algorithm"[Title/Abstract:~4] OR "decision algorithms"[Title/Abstract:~4] OR "decision process"[Title/Abstract:~4] OR "decision processes"[Title/Abstract:~4] OR "decision method"[Title/Abstract:~4] OR "decision methods"[Title/Abstract:~4] OR "decision intervention"[Title/Abstract:~4] OR "decision interventions"[Title/Abstract:~4] OR "decision material"[Title/Abstract:~4] OR "decision materials"[Title/Abstract:~4] OR "interactive tool"[Title/Abstract:~4] OR "interactive tools"[Title/Abstract:~4] OR "interaction tool"[Title/Abstract:~4] OR "interaction tools"[Title/Abstract:~4] |
| 10 | "interactive internet"[Title/Abstract] OR "interactive online"[Title/Abstract] OR "interactive graphic*"[Title/Abstract] OR "interactive booklet*"[Title/Abstract] OR "choice behavior"[Title/Abstract] OR "choice behaviour"[Title/Abstract] OR "informed choice*"[Title/Abstract] OR "informed decision*"[Title/Abstract] OR "decision board*"[Title/Abstract] OR "decision guide*"[Title/Abstract] OR "decision counseling"[Title/Abstract] OR "decision counselling"[Title/Abstract] OR "risk communication tool*"[Title/Abstract] OR "risk communication method*"[Title/Abstract] OR "risk assessment tool*"[Title/Abstract] OR "risk assessment method*"[Title/Abstract] OR "risk information tool*"[Title/Abstract] OR "computer* decision making"[Title/Abstract] OR "interactive health communication"[Title/Abstract] OR "shared decision making"[Title/Abstract] OR "interactive risk communication"[Title/Abstract] |
| 11 | (((((((("Decision Support Techniques"[Mesh]) OR "Decision Making"[Mesh:NoExp]) OR "Choice Behavior"[Mesh:NoExp]) OR "Decision Support Systems, Clinical"[Mesh]) OR "Decision Making, Computer-Assisted"[Mesh]) OR "Decision Making, Shared"[Mesh]) OR "Decision Trees"[Mesh]) OR "Patient Education as Topic/methods"[Mesh]) OR "Health Education/methods"[Mesh] |
| **12** | **9 OR 10 OR 11**  (("decision aid"[Title/Abstract:~4] OR "decision aids"[Title/Abstract:~4] OR "decision tool"[Title/Abstract:~4] OR "decision tools"[Title/Abstract:~4] OR "decision support"[Title/Abstract:~4] OR "decision making"[Title/Abstract:~3] OR "decision instrument"[Title/Abstract:~4] OR "decision instruments" [Title/Abstract:~4] OR "decision technology"[Title/Abstract:~4] OR "decision technologies"[Title/Abstract:~4] OR "decision technique"[Title/Abstract:~4] OR "decision techniques"[Title/Abstract:~4] OR "decision system"[Title/Abstract:~4] OR "decision systems"[Title/Abstract:~4] OR "decision program"[Title/Abstract:~4] OR "decision programs"[Title/Abstract:~4] OR "decision programme"[Title/Abstract:~4] OR "decision programmes"[Title/Abstract:~4] OR "decision algorithm"[Title/Abstract:~4] OR "decision algorithms"[Title/Abstract:~4] OR "decision process"[Title/Abstract:~4] OR "decision processes"[Title/Abstract:~4] OR "decision method"[Title/Abstract:~4] OR "decision methods"[Title/Abstract:~4] OR "decision intervention"[Title/Abstract:~4] OR "decision interventions"[Title/Abstract:~4] OR "decision material"[Title/Abstract:~4] OR "decision materials"[Title/Abstract:~4] OR "interactive tool"[Title/Abstract:~4] OR "interactive tools"[Title/Abstract:~4] OR "interaction tool"[Title/Abstract:~4] OR "interaction tools"[Title/Abstract:~4]) OR ((((((((("Decision Support Techniques"[Mesh]) OR "Decision Making"[Mesh:NoExp]) OR "Choice Behavior"[Mesh:NoExp]) OR "Decision Support Systems, Clinical"[Mesh]) OR "Decision Making, Computer-Assisted"[Mesh]) OR "Decision Making, Shared"[Mesh]) OR "Decision Trees"[Mesh]) OR "Patient Education as Topic/methods"[Mesh]) OR "Health Education/methods"[Mesh])) OR ("interactive internet"[Title/Abstract] OR "interactive online"[Title/Abstract] OR "interactive graphic*"[Title/Abstract] OR "interactive booklet*"[Title/Abstract] OR "choice behavior"[Title/Abstract] OR "choice behaviour"[Title/Abstract] OR "informed choice*"[Title/Abstract] OR "informed decision*"[Title/Abstract] OR "decision board*"[Title/Abstract] OR "decision guide*"[Title/Abstract] OR "decision counseling"[Title/Abstract] OR "decision counselling"[Title/Abstract] OR "risk communication tool*"[Title/Abstract] OR "risk communication method*"[Title/Abstract] OR "risk assessment tool*"[Title/Abstract] OR "risk assessment method*"[Title/Abstract] OR "risk information tool*"[Title/Abstract] OR "computer* decision making"[Title/Abstract] OR "interactive health communication"[Title/Abstract] OR "shared decision making"[Title/Abstract] OR "interactive risk communication"[Title/Abstract]) |
| **13** | **5 or 8**  (((((tenofovir alafenamide[Title/Abstract] AND emtricitabine[Title/Abstract]) OR (((("tenofovir disoproxil fumarate"[Title/Abstract]) AND (emtricitabine[Title/Abstract])))))) OR ("HIV Infections/prevention and control"[Mesh] OR "HIV Infections/transmission"[Mesh] )) OR ( "Acquired Immunodeficiency Syndrome/prevention and control"[Mesh] OR "Acquired Immunodeficiency Syndrome/transmission"[Mesh] )) OR "HIV Seropositivity/transmission"[Mesh]) OR "HIV Testing"[Mesh]) OR "Anti-HIV Agents"[Mesh]) OR "Emtricitabine, Tenofovir Disoproxil Fumarate Drug Combination"[Mesh]) OR "Pre-Exposure Prophylaxis"[Mesh]) OR "Post-Exposure Prophylaxis"[Mesh]) OR "Circumcision, Male"[Mesh]) OR "cabotegravir" [Supplementary Concept]) OR "lenacapavir" [Supplementary Concept]) OR "tenofovir alafenamide" [Supplementary Concept]) OR "Dapivirine" [Supplementary Concept])))) OR ("pre-exposure prophylaxis"[Title/Abstract] OR "post-exposure prophylaxis"[Title/Abstract] OR "traditional circumcision"[Title/Abstract] OR PrEP[Title/Abstract] OR PEP[Title/Abstract] OR PEPSE[Title/Abstract] OR VMMC[Title/Abstract] OR CAB-LA[Title/Abstract] OR TDF/FTC[Title/Abstract] OR "tenofovir alafenamide"[Title/Abstract] OR F/TAF[Title/Abstract] OR HIVST[Title/Abstract])))) OR (((((((((("HIV TRI-DOT"[Title/Abstract]) OR ("CABENUVA"[Title/Abstract])) OR ("Isentress"[Title/Abstract])) OR ("OraQuick"[Title/Abstract])) OR ("INSTI"[Title/Abstract])) OR ("TRUVADA"[Title/Abstract])) OR ("Apretude"[Title/Abstract])) OR (Determine HIV-1/2[Title/Abstract])) OR ("ChemBio"[Title/Abstract])) OR (Sunlenca[Title/Abstract])))) OR ("HIV prevention"[Title/Abstract:~5] OR "male circumcision"[Title/Abstract:~3] OR "circumcised male"[Title/Abstract:~3] OR "circumcised males"[Title/Abstract:~3] OR "long-acting cabotegravir"[Title/Abstract:~2] OR "injectible cabotegravir"[Title/Abstract:~3] OR "cabotegravir injection"[Title/Abstract:~3] OR "cabotegravir injections"[Title/Abstract:~3] OR "long-acting lenacapavir"[Title/Abstract:~2] OR "lenacapavir injection"[Title/Abstract:~3] OR "lenacapavir injections"[Title/Abstract:~3] OR "Dapivirine ring"[Title/Abstract:~3] OR "HIV test"[Title/Abstract:~2] OR "HIV tests"[Title/Abstract:~2] OR "HIV tested"[Title/Abstract:~2] OR "HIV testing"[Title/Abstract:~2] OR "HIV screen"[Title/Abstract:~2] OR "HIV screened"[Title/Abstract:~2] OR "HIV screening"[Title/Abstract:~2])) OR (((((((((("Contraception"[Mesh]) OR "Contraceptive Agents"[Mesh]) OR "Family Planning Services"[Mesh]) OR "Contraceptive Devices"[Mesh]) OR "Vasectomy"[Mesh]) OR "Sterilization, Tubal"[Mesh]) OR "Medroxyprogesterone Acetate"[Mesh]) OR "norethindrone enanthate" [Supplementary Concept]) OR (contracepti*[Title/Abstract] OR "birth control"[Title/Abstract] OR "fertility control"[Title/Abstract] OR "progest* only"[Title/Abstract] OR "mini pill"[Title/Abstract] OR "combination pill"[Title/Abstract] OR "family planning"[Title/Abstract] OR condom*[Title/Abstract] OR "intrauterine device*"[Title/Abstract] OR IUD[Title/Abstract] OR IUDs[Title/Abstract] OR "vaginal ring*"[Title/Abstract] OR "vaginal sponge*"[Title/Abstract] OR "vaginal shield*"[Title/Abstract] OR "vaginal diaphragm*"[Title/Abstract] OR "cervical cap*"[Title/Abstract] OR Jadelle[Title/Abstract] OR Implanon[Title/Abstract] OR Nexplanon[Title/Abstract] OR "levonorgestrel implant*"[Title/Abstract] OR "etonogestrel implant*"[Title/Abstract] OR NuvaRing[Title/Abstract] OR Annovera[Title/Abstract] OR EluRyng[Title/Abstract] OR LARC[Title/Abstract] OR "depot medroxyprogesterone acetate"[Title/Abstract] OR Depo-Provera[Title/Abstract] OR DMPA[Title/Abstract] OR "Depo-SubQ Provera 104"[Title/Abstract] OR "medroxyprogesterone acetate"[Title/Abstract] OR "Sayana Press"[Title/Abstract] OR "norethisterone enanthate"[Title/Abstract] OR "norethindrone enanthate"[Title/Abstract] OR Levonelle[Title/Abstract] OR "morning after pill*"[Title/Abstract] OR "rhythm method"[Title/Abstract] OR "withdrawal method"[Title/Abstract] OR "coitus interruptus"[Title/Abstract] OR "ovulation method"[Title/Abstract] OR "standard days method"[Title/Abstract] OR "calendar method"[Title/Abstract] OR "lactational amenorrhea method"[Title/Abstract] OR "sexual abstinence"[Title/Abstract] OR "periodic abstinence"[Title/Abstract] OR "male sterilisation*"[Title/Abstract] OR "female sterilisation*"[Title/Abstract] OR "male sterilization*"[Title/Abstract] OR "female sterilization*"[Title/Abstract] OR vasectomy[Title/Abstract] OR "vas ligation*"[Title/Abstract] OR "tubal ligation*") OR "Drospirenone"[Title/Abstract] OR "Azurette"[Title/Abstract] OR "Microgynon"[Title/Abstract] OR "Aviane"[Title/Abstract] OR "Desogestrel"[Title/Abstract] OR "Lessina"[Title/Abstract] OR "Levora"[Title/Abstract] OR "Loestrin"[Title/Abstract] OR "Lutera"[Title/Abstract] OR "rigevidon" [Title/Abstract] OR "Apri" [Title/Abstract] OR "colique"[Title/Abstract] OR "Cilique"[Title/Abstract] OR "Lo/ovral-28"[Title/Abstract] OR "Ortho-Novum"[Title/Abstract] OR "Portia"[Title/Abstract] OR "Drospirenone/Ethinyl estradiol"[Title/Abstract] OR "Alesse"[Title/Abstract] OR "Levonorgestrel"[Title/Abstract] OR "Natazia"[Title/Abstract] OR "ortho tri-cyclen"[Title/Abstract] OR "nuvaring"[Title/Abstract] OR "annovera"[Title/Abstract] OR "EluRyng"[Title/Abstract] OR "etonogestrel/ ethynyl estradiol"[Title/Abstract] OR "segestrone acetate/ ethinyl estradiol"[Title/Abstract] OR "norlevo"[Title/Abstract] OR "postinor"[Title/Abstract] OR "Noriday"[Title/Abstract] OR "Xulane"[Title/Abstract] OR "Twirla"[Title/Abstract] OR "zafemy"[Title/Abstract] OR "Evra"[Title/Abstract] OR "Ortho Evra"[Title/Abstract] OR "Mirena"[Title/Abstract] OR "Liletta"[Title/Abstract] OR "Kyleena"[Title/Abstract] OR "Skyla"[Title/Abstract] OR "Paragard"[Title/Abstract])) OR "Copper T380A"[Title/Abstract])) OR "LNg 20"[Title/Abstract]) OR "Nova T"[Title/Abstract] OR "spermicide"[Title/Abstract] OR "condom"[Title/Abstract] OR "symptothermal"[Title/Abstract] OR "post ovulation coitus*"[Title/Abstract] OR "periodic abstinence"[Title/Abstract] OR "vaginal douche"[Title/Abstract] OR "option 2"[Title/Abstract] OR "unwanted pregcard"[Title/Abstract] OR "after a"[Title/Abstract] OR "unwanted72"[Title/Abstract])) OR "plan b"[Title/Abstract] OR "Ella one"[Title/Abstract] OR "Ellaone"[Title/Abstract] OR "My choice"[Title/Abstract] OR "take action"[Title/Abstract] OR "after pill"[Title/Abstract] OR "afterpill" [Title/Abstract] OR "herstyle"[Title/Abstract] OR "her style"[Title/Abstract] OR "opcicon onestep"[Title/Abstract] OR "opcicon one step"[Title/Abstract] OR "Plan B"[Title/Abstract] OR "Ulipristal"[Title/Abstract] OR "Next Choice"[Title/Abstract])) OR "New Day"[Title/Abstract] OR "Ortho All-flex diaphragm"[Title/Abstract] OR "Cap diaphragm"[Title/Abstract] OR "Milex"[Title/Abstract] OR "Caya"[Title/Abstract] OR "Postinor"[Title/Abstract] OR "Escapelle"[Title/Abstract] OR "Cerazette"[Title/Abstract] OR "Yasmin"[Title/Abstract] OR "Norgeston"[Title/Abstract] OR "Marvelon"[Title/Abstract] OR "Femilion"[Title/Abstract] OR "Norlutin"[Title/Abstract] OR "Novafem"[Title/Abstract] OR "Mesigyna"[Title/Abstract])) OR "Lunelle"[Title/Abstract] OR "feminena"[Title/Abstract] OR "Cyclofem"[Title/Abstract] OR "Noristerat"[Title/Abstract] OR "NorLevo"[Title/Abstract])) |
| **14** | **12 AND 13**  ((((((tenofovir alafenamide[Title/Abstract] AND emtricitabine[Title/Abstract]) OR (((("tenofovir disoproxil fumarate"[Title/Abstract]) AND (emtricitabine[Title/Abstract])))))) OR ("HIV Infections/prevention and control"[Mesh] OR "HIV Infections/transmission"[Mesh] )) OR ( "Acquired Immunodeficiency Syndrome/prevention and control"[Mesh] OR "Acquired Immunodeficiency Syndrome/transmission"[Mesh] )) OR "HIV Seropositivity/transmission"[Mesh]) OR "HIV Testing"[Mesh]) OR "Anti-HIV Agents"[Mesh]) OR "Emtricitabine, Tenofovir Disoproxil Fumarate Drug Combination"[Mesh]) OR "Pre-Exposure Prophylaxis"[Mesh]) OR "Post-Exposure Prophylaxis"[Mesh]) OR "Circumcision, Male"[Mesh]) OR "cabotegravir" [Supplementary Concept]) OR "lenacapavir" [Supplementary Concept]) OR "tenofovir alafenamide" [Supplementary Concept]) OR "Dapivirine" [Supplementary Concept])))) OR ("pre-exposure prophylaxis"[Title/Abstract] OR "post-exposure prophylaxis"[Title/Abstract] OR "traditional circumcision"[Title/Abstract] OR PrEP[Title/Abstract] OR PEP[Title/Abstract] OR PEPSE[Title/Abstract] OR VMMC[Title/Abstract] OR CAB-LA[Title/Abstract] OR TDF/FTC[Title/Abstract] OR "tenofovir alafenamide"[Title/Abstract] OR F/TAF[Title/Abstract] OR HIVST[Title/Abstract])))) OR (((((((((("HIV TRI-DOT"[Title/Abstract]) OR ("CABENUVA"[Title/Abstract])) OR ("Isentress"[Title/Abstract])) OR ("OraQuick"[Title/Abstract])) OR ("INSTI"[Title/Abstract])) OR ("TRUVADA"[Title/Abstract])) OR ("Apretude"[Title/Abstract])) OR (Determine HIV-1/2[Title/Abstract])) OR ("ChemBio"[Title/Abstract])) OR (Sunlenca[Title/Abstract])))) OR ("HIV prevention"[Title/Abstract:~5] OR "male circumcision"[Title/Abstract:~3] OR "circumcised male"[Title/Abstract:~3] OR "circumcised males"[Title/Abstract:~3] OR "long-acting cabotegravir"[Title/Abstract:~2] OR "injectable cabotegravir"[Title/Abstract:~3] OR "cabotegravir injection"[Title/Abstract:~3] OR "cabotegravir injections"[Title/Abstract:~3] OR "long-acting lenacapavir"[Title/Abstract:~2] OR "lenacapavir injection"[Title/Abstract:~3] OR "lenacapavir injections"[Title/Abstract:~3] OR "Dapivirine ring"[Title/Abstract:~3] OR "HIV test"[Title/Abstract:~2] OR "HIV tests"[Title/Abstract:~2] OR "HIV tested"[Title/Abstract:~2] OR "HIV testing"[Title/Abstract:~2] OR "HIV screen"[Title/Abstract:~2] OR "HIV screened"[Title/Abstract:~2] OR "HIV screening"[Title/Abstract:~2])) OR (((((((((("Contraception"[Mesh]) OR "Contraceptive Agents"[Mesh]) OR "Family Planning Services"[Mesh]) OR "Contraceptive Devices"[Mesh]) OR "Vasectomy"[Mesh]) OR "Sterilization, Tubal"[Mesh]) OR "Medroxyprogesterone Acetate"[Mesh]) OR "norethindrone enanthate" [Supplementary Concept]) OR (contracepti*[Title/Abstract] OR "birth control"[Title/Abstract] OR "fertility control"[Title/Abstract] OR "progest* only"[Title/Abstract] OR "mini pill"[Title/Abstract] OR "combination pill"[Title/Abstract] OR "family planning"[Title/Abstract] OR condom*[Title/Abstract] OR "intrauterine device*"[Title/Abstract] OR IUD[Title/Abstract] OR IUDs[Title/Abstract] OR "vaginal ring*"[Title/Abstract] OR "vaginal sponge*"[Title/Abstract] OR "vaginal shield*"[Title/Abstract] OR "vaginal diaphragm*"[Title/Abstract] OR "cervical cap*"[Title/Abstract] OR Jadelle[Title/Abstract] OR Implanon[Title/Abstract] OR Nexplanon[Title/Abstract] OR "levonorgestrel implant*"[Title/Abstract] OR "etonogestrel implant*"[Title/Abstract] OR NuvaRing[Title/Abstract] OR Annovera[Title/Abstract] OR EluRyng[Title/Abstract] OR LARC[Title/Abstract] OR "depot medroxyprogesterone acetate"[Title/Abstract] OR Depo-Provera[Title/Abstract] OR DMPA[Title/Abstract] OR "Depo-SubQ Provera 104"[Title/Abstract] OR "medroxyprogesterone acetate"[Title/Abstract] OR "Sayana Press"[Title/Abstract] OR "norethisterone enanthate"[Title/Abstract] OR "norethindrone enanthate"[Title/Abstract] OR Levonelle[Title/Abstract] OR "morning after pill*"[Title/Abstract] OR "rhythm method"[Title/Abstract] OR "withdrawal method"[Title/Abstract] OR "coitus interruptus"[Title/Abstract] OR "ovulation method"[Title/Abstract] OR "standard days method"[Title/Abstract] OR "calendar method"[Title/Abstract] OR "lactational amenorrhea method"[Title/Abstract] OR "sexual abstinence"[Title/Abstract] OR "periodic abstinence"[Title/Abstract] OR "male sterilisation*"[Title/Abstract] OR "female sterilisation*"[Title/Abstract] OR "male sterilization*"[Title/Abstract] OR "female sterilization*"[Title/Abstract] OR vasectomy[Title/Abstract] OR "vas ligation*"[Title/Abstract] OR "tubal ligation*") OR "Drospirenone"[Title/Abstract] OR "Azurette"[Title/Abstract] OR "Microgynon"[Title/Abstract] OR "Aviane"[Title/Abstract] OR "Desogestrel"[Title/Abstract] OR "Lessina"[Title/Abstract] OR "Levora"[Title/Abstract] OR "Loestrin"[Title/Abstract] OR "Lutera"[Title/Abstract] OR "rigevidon" [Title/Abstract] OR "Apri" [Title/Abstract] OR "colique"[Title/Abstract] OR "Cilique"[Title/Abstract] OR "Lo/ovral-28"[Title/Abstract] OR "Ortho-Novum"[Title/Abstract] OR "Portia"[Title/Abstract] OR "Drospirenone/Ethinyl estradiol"[Title/Abstract] OR "Alesse"[Title/Abstract] OR "Levonorgestrel"[Title/Abstract] OR "Natazia"[Title/Abstract] OR "ortho tri-cyclen"[Title/Abstract] OR "nuvaring"[Title/Abstract] OR "annovera"[Title/Abstract] OR "EluRyng"[Title/Abstract] OR "etonogestrel/ ethynyl estradiol"[Title/Abstract] OR "segestrone acetate/ ethinyl estradiol"[Title/Abstract] OR "norlevo"[Title/Abstract] OR "postinor"[Title/Abstract] OR "Noriday"[Title/Abstract] OR "Xulane"[Title/Abstract] OR "Twirla"[Title/Abstract] OR "zafemy"[Title/Abstract] OR "Evra"[Title/Abstract] OR "Ortho Evra"[Title/Abstract] OR "Mirena"[Title/Abstract] OR "Liletta"[Title/Abstract] OR "Kyleena"[Title/Abstract] OR "Skyla"[Title/Abstract] OR "Paragard"[Title/Abstract])) OR "Copper T380A"[Title/Abstract])) OR "LNg 20"[Title/Abstract]) OR "Nova T"[Title/Abstract] OR "spermicide"[Title/Abstract] OR "condom"[Title/Abstract] OR "symptothermal"[Title/Abstract] OR "post ovulation coitus*"[Title/Abstract] OR "periodic abstinence"[Title/Abstract] OR "vaginal douche"[Title/Abstract] OR "option 2"[Title/Abstract] OR "unwanted pregcard"[Title/Abstract] OR "after a"[Title/Abstract] OR "unwanted72"[Title/Abstract])) OR "plan b"[Title/Abstract] OR "Ella one"[Title/Abstract] OR "Ellaone"[Title/Abstract] OR "My choice"[Title/Abstract] OR "take action"[Title/Abstract] OR "after pill"[Title/Abstract] OR "afterpill" [Title/Abstract] OR "herstyle"[Title/Abstract] OR "her style"[Title/Abstract] OR "opcicon onestep"[Title/Abstract] OR "opcicon one step"[Title/Abstract] OR "Plan B"[Title/Abstract] OR "Ulipristal"[Title/Abstract] OR "Next Choice"[Title/Abstract])) OR "New Day"[Title/Abstract] OR "Ortho All-flex diaphragm"[Title/Abstract] OR "Cap diaphragm"[Title/Abstract] OR "Milex"[Title/Abstract] OR "Caya"[Title/Abstract] OR "Postinor"[Title/Abstract] OR "Escapelle"[Title/Abstract] OR "Cerazette"[Title/Abstract] OR "Yasmin"[Title/Abstract] OR "Norgeston"[Title/Abstract] OR "Marvelon"[Title/Abstract] OR "Femilion"[Title/Abstract] OR "Norlutin"[Title/Abstract] OR "Novafem"[Title/Abstract] OR "Mesigyna"[Title/Abstract])) OR "Lunelle"[Title/Abstract] OR "feminena"[Title/Abstract] OR "Cyclofem"[Title/Abstract] OR "Noristerat"[Title/Abstract] OR "NorLevo"[Title/Abstract]))) AND ((("decision aid"[Title/Abstract:~4] OR "decision aids"[Title/Abstract:~4] OR "decision tool"[Title/Abstract:~4] OR "decision tools"[Title/Abstract:~4] OR "decision support"[Title/Abstract:~4] OR "decision making"[Title/Abstract:~3] OR "decision instrument"[Title/Abstract:~4] OR "decision instruments" [Title/Abstract:~4] OR "decision technology"[Title/Abstract:~4] OR "decision technologies"[Title/Abstract:~4] OR "decision technique"[Title/Abstract:~4] OR "decision techniques"[Title/Abstract:~4] OR "decision system"[Title/Abstract:~4] OR "decision systems"[Title/Abstract:~4] OR "decision program"[Title/Abstract:~4] OR "decision programs"[Title/Abstract:~4] OR "decision programme"[Title/Abstract:~4] OR "decision programmes"[Title/Abstract:~4] OR "decision algorithm"[Title/Abstract:~4] OR "decision algorithms"[Title/Abstract:~4] OR "decision process"[Title/Abstract:~4] OR "decision processes"[Title/Abstract:~4] OR "decision method"[Title/Abstract:~4] OR "decision methods"[Title/Abstract:~4] OR "decision intervention"[Title/Abstract:~4] OR "decision interventions"[Title/Abstract:~4] OR "decision material"[Title/Abstract:~4] OR "decision materials"[Title/Abstract:~4] OR "interactive tool"[Title/Abstract:~4] OR "interactive tools"[Title/Abstract:~4] OR "interaction tool"[Title/Abstract:~4] OR "interaction tools"[Title/Abstract:~4]) OR ((((((((("Decision Support Techniques"[Mesh]) OR "Decision Making"[Mesh:NoExp]) OR "Choice Behavior"[Mesh:NoExp]) OR "Decision Support Systems, Clinical"[Mesh]) OR "Decision Making, Computer-Assisted"[Mesh]) OR "Decision Making, Shared"[Mesh]) OR "Decision Trees"[Mesh]) OR "Patient Education as Topic/methods"[Mesh]) OR "Health Education/methods"[Mesh])) OR ("interactive internet"[Title/Abstract] OR "interactive online"[Title/Abstract] OR "interactive graphic*"[Title/Abstract] OR "interactive booklet*"[Title/Abstract] OR "choice behavior"[Title/Abstract] OR "choice behaviour"[Title/Abstract] OR "informed choice*"[Title/Abstract] OR "informed decision*"[Title/Abstract] OR "decision board*"[Title/Abstract] OR "decision guide*"[Title/Abstract] OR "decision counseling"[Title/Abstract] OR "decision counselling"[Title/Abstract] OR "risk communication tool*"[Title/Abstract] OR "risk communication method*"[Title/Abstract] OR "risk assessment tool*"[Title/Abstract] OR "risk assessment method*"[Title/Abstract] OR "risk information tool*"[Title/Abstract] OR "computer* decision making"[Title/Abstract] OR "interactive health communication"[Title/Abstract] OR "shared decision making"[Title/Abstract] OR "interactive risk communication"[Title/Abstract])) |
