# Supplementary material for: Availability, acceptability and adoption of decision aids for HIV prevention and contraception for young people: a scoping review protocol
Source: BMJ Open. 2026 Mar 4;16(3):e106381. doi: 10.1136/bmjopen-2025-106381 (PMC12970088; doi:10.1136/bmjopen-2025-106381)
Supplement: online supplemental file 2 [file bmjopen-16-3-s002.docx]

**APPENDIX 2: TOOL FOR ASSESSING SUITABILITY OF HIV PREVENTION AND CONTRACEPTION DECISION AIDS FOR YOUNG PEOPLE**

To inform the development or adaptation of context-relevant decision aids for HIV prevention and contraception, we are conducting a scoping review to explore the availability, acceptability, and use of decision aids for HIV prevention and contraception for young people. This tool is designed to assess the accessibility, appropriateness, acceptability, equitability, and effectiveness of available HIV prevention and contraception decision aids for young people according to the WHO Quality Assessment Guidebook: A guide to assessing health services for adolescent clients standards**.**

# **DOMAIN 1: SCOPE AND PURPOSE**

1. What is the overall objective of the decision aid:

| **Category** | **Details** |
| --- | --- |
| **Population** |  |
| **Concept** |  |
| **Context** |  |

1. When was the decision aid designed? Month/Year_ _____________________
2. Has this decision aid been updated? **Yes**  **No** ⬜

if yes, what is the name of updated version **_______________________________________________________________________________________________________________________________________________________________________________**

1. What is the format of the decision aid ⬜ **Print**   **Digital**
2. Is the decision aid:

**patient centred/facing** *(can be used by the patient/user independently)*

⬜ **health worker centred/facing alone** *(cannot be used by the patient/user independently, HW giving information to the client)*

⬜ **health worker centred/facing with client engagement** *(cannot be used by the patient independently, HW using the decision aid together with client)*

⬜ **both** *(can be used by patients/users or health worker independently)*

1. Which individuals were involved in the development process of the decision aid?

| **Category of participants** | **Tick where applicable** | **Role in the decision aid development** |
| --- | --- | --- |
| Policy makers/ MoH |  |  |
| Communities |  |  |
| Men |  |  |
| Women |  |  |
| Young people |  |  |
| Health workers |  |  |
| Other |  |  |

1. What are the expected benefits of using the decision aid, as described by the authors?
2. Was alpha testing of the decision aid done? **Yes ⬜ No**

If yes, among who?

Communities **⬜**

Health workers **⬜**

Technical staff **⬜**

Other **⬜**

9. Were the views of those that participated in alpha testing incorporated **Yes** ⬜ **No**

10. Was beta testing of the decision aid conducted  **Yes ⬜ No ⬜**

If yes, among who?

Communities **⬜**

Health workers **⬜**

Technical staff **⬜**

Other **⬜**

11. Were the views of those that participated in beta testing incorporated **Yes** ⬜ **No ⬜**

**DOMAIN 2: ACCESSIBLE: Adolescents are able to obtain the health services that are available**

1. Did the authors report the cost of accessing the decision aid by young people?

**Yes** ⬜ **No Not reported** ⬜ **Not applicable**

1. Did young people fail to access the decision aid because of costs associated with accessing the decision aid as reported by authors?

⬜ **Yes No Not reported**  ⬜ **Not applicable**

1. Were the young people (especially younger ones, 15-19 years) allowed access to the decision aid? **Yes** ⬜ **No Not reported**  ⬜ **Not applicable**
2. Were single (unmarried) people allowed access to the decision aid?

**Yes** ⬜ **No Not reported**  ⬜ **Not applicable**

1. Can this decision aid be considered conveniently located to young people (easy to access)? **Yes No** ⬜ **Not reported**  ⬜ **Not applicable**
2. Can this decision aid be provided to young people at no cost?

⬜ **Yes No Not reported**  ⬜ **Not applicable**

1. Can this decision aid be provided to young people on multiple access locations?

**Yes** ⬜ **No** ⬜ **Not reported**  ⬜ **Not applicable**

1. Can the decision aid be provided to young people at flexible times (as and when needed by young people)  **Yes** ⬜ **No** ⬜ **Not reported**  ⬜ **Not applicable**
2. Does the decision aid make young people aware of available HIV prevention or contraception services (depending on type of decision aid)?

**Yes** ⬜ **Partial** ⬜ **No** ⬜ **Not reported**  ⬜ **Not applicable**

1. Does the decision aid provide awareness on where to access HIV prevention or contraception services?

**Yes** ⬜ **No** ⬜ **Not reported**  ⬜ **Not applicable**

1. Does the decision aid provide awareness of how to access available HIV prevention or contraception services?

**Yes** ⬜ **No** ⬜ **Not reported**  ⬜ **Not applicable**

**What are the important considerations or aspects of this decision aid that make it accessible to young people?**

**What are the important considerations or aspects not met by this decision aid that make it inaccessible to young people?**

**Considering the important aspects met and not met by this decision aid, what is the overall view on accessibility of the decision aid for young people?**

**DOMAIN 3: ACCEPTABLE: Adolescents are willing to obtain the health services that are available**

1. Did the authors report acceptability of the decision aid to young people

**Yes** ⬜ **No** ⬜ **Not reported** ⬜ **Not applicable**

1. Did the young people find the decision aid acceptable as reported by the authors?

**Yes** ⬜ **No** ⬜ **Not reported** ⬜ **Not applicable**

1. Is the decision aid written in a way that feels encouraging and not judgemental?

**Yes** ⬜ **No** ⬜ **Not reported** ⬜ **Not applicable**

1. Can young people access the decision aid privately?

**Yes** ⬜ **No Not reported** ⬜ **Not applicable**

1. Does the decision aid protect young people’s personal information

**Yes** ⬜ **No** ⬜ **Not reported** ⬜ **Not applicable**

1. Is the decision aid designed in a way that allows young people to find and read the information they need quickly?

**Yes** ⬜ **No** ⬜ **Not reported** ⬜ **Not applicable**

**What are the important considerations or aspects of this decision aid that make it acceptable to young people?**

**What are the important considerations or aspects not met by this decision aid that make unacceptable to young people?**

**-**

**Considering the important aspects met and not met by this decision aid, what is the overall acceptability of the decision aid to young people?**

**DOMAIN 4: APPROPRIATE: The right health services (i.e. the ones they need) are provided to them**

1. Did the authors report appropriateness of the decision aid to young people

**Yes** ⬜ **No** ⬜ **Not reported** ⬜ **Not applicable**

1. Did the young people report finding the decision aid appropriate?

**Yes** ⬜ **No** ⬜ **Not reported** ⬜ **Not applicable**

1. Does the decision aid provide young people with the information they need about HIV prevention or contraception?
2. HIV prevention or contraception options

**All** ⬜ **Some** ⬜ **None** ⬜ **Not reported** ⬜ **Not applicable**

1. HIV prevention or contraception effectiveness

**All** ⬜ **Some** ⬜ **None** ⬜ **Not reported** ⬜ **Not applicable**

1. HIV prevention or contraception side effects/risks

**All** ⬜ **Some** ⬜ **None**  ⬜ **Not reported** ⬜ **Not applicable**

1. HIV prevention or contraception advantages

**All** ⬜ **Some** ⬜ **None** ⬜ **Not reported** ⬜ **Not applicable**

1. HIV prevention or contraception disadvantages

**All** ⬜ **Some** ⬜ **None** ⬜ **Not reported** ⬜ **Not applicable**

1. Is the decision aid visually appealing to young people (light text, use of pictorials, use of graphics, effective use of colour)?

**Yes** ⬜ **No** ⬜ **Not reported** ⬜ **Not applicable**

1. Does the decision aid factor in patient values and preferences?

**Yes** ⬜ **No** ⬜ **Not reported** ⬜ **Not applicable**

1. Is the decision aid interactive in use (allow for preferences to influence choices presented)? **Yes** ⬜ **No** ⬜ **Not reported** ⬜ **Not applicable**
2. Is the information in the decision aid based on scientific evidence to allow informed decision making by young people?

**Yes** ⬜ **No** ⬜ **Not reported** ⬜ **Not applicable**

1. Does the decision aid provide ease of use/navigation by young people (instructions for use are provided)?  **Yes** ⬜ **No** ⬜ **Not reported** ⬜ **Not applicable**
2. Does the decision aid include instructions on how young people can access support from health workers if they need help?

**Yes No** ⬜ **Not reported** ⬜ **Not applicable**

1. Does the decision aid provide information on where to access additional information if needed by young people? **Yes** ⬜ **No** ⬜ **Not reported** ⬜ **Not applicable**

**What are the important considerations or aspects of this decision aid that make it appropriate to young people?**

**What are the important considerations or aspects not met by this decision aid that make it inappropriate to young people?**

**Considering the important aspects met and not met by this decision aid, what is the overall appropriateness of the decision aid to young people?**

**DOMAIN 5: EFFECTIVE: The right health services are provided in the right way and make a positive contribution to their health.**

1. Did the authors report effectiveness of the decision aid to young people?

**Yes** ⬜ **Not reported** ⬜ **Not applicable**

1. Did the young people find the decision aid effective in supporting decision making or in improving any reported outcome?

**Yes** ⬜ **No Not reported**  ⬜ **Not applicable**

1. Does the research/program data show effectiveness of the decision aid in improving any important outcomes? **Yes** ⬜ **No** ⬜ **Not reported** ⬜ **Not applicable**

**What are the important considerations or aspects of this decision aid that make it effective to young people?**

**What are the important considerations or aspects not met by this decision aid that make it ineffective to young people?**

**Considering the important aspects met and not met by this decision aid, what is the overall effectiveness of the decision aid to young people?**

**DOMAIN 6: EQUITABLE: All adolescents, not just selected groups, are able to obtain the health services that are available.**

1. Did the authors report equitability of the decision aid to young people?

⬜ **Yes** 🗙**No** ⬜ **Not reported** ⬜ **Not applicable**

1. Did the young people report finding the decision aid equitable?

⬜ **Yes No** 🗙 **Not reported** ⬜ **Not applicable**

1. Does the format (print or digital) of the decision aid make it accessible to young people of different:

Age: ⬜ **Yes** ⬜ **No** ⬜ **Not reported** ⬜ **Not applicable**

Sex/Gender: ⬜ **Yes** ⬜ **No** ⬜ **Not reported** ⬜ **Not applicable**

Ethnicity: ⬜ **Yes** ⬜ **No** ⬜ **Not reported** ⬜ **Not applicable**

Social status: ⬜ **Yes** ⬜ **No** ⬜ **Not reported** ⬜ **Not applicable**

Disability: ⬜ **Yes** ⬜ **No** ⬜ **Not reported** ⬜ **Not applicable**

Literacy: ⬜ **Yes** ⬜ **No** ⬜ **Not reported** ⬜ **Not applicable**

Occupation: ⬜ **Yes** ⬜ **No** ⬜ **Not reported** ⬜ **Not applicable**

(incl vulnerable groups such as sex workers)

1. Can the decision aid be provided on platforms/locations (health facility, outreach, online) accessible to young people of different:

Age: ⬜ **Yes** ⬜ **No** ⬜ **Not reported** ⬜ **Not applicable**

Sex /Gender: ⬜ **Yes** ⬜ **No** ⬜ **Not reported** ⬜ **Not applicable**

Ethnicity: ⬜ **Yes** ⬜ **No** ⬜ **Not reported** ⬜ **Not applicable**

Social status: ⬜ **Yes** ⬜ **No** ⬜ **Not reported** ⬜ **Not applicable**

Disability: ⬜ **Yes** ⬜ **No** ⬜ **Not reported** ⬜ **Not applicable**

Literacy: ⬜ **Yes** ⬜ **No** ⬜ **Not reported** ⬜ **Not applicable**

Occupation: ⬜ **Yes** ⬜ **No** ⬜ **Not reported** ⬜ **Not applicable**

(incl vulnerable groups such as sex workers)

1. Is the HIV prevention and contraception information on decision aid appropriate to young people of different:

Age: ⬜ **Yes** ⬜ **No** ⬜ **Not reported** ⬜ **Not applicable**

Sex /Gender: ⬜ **Yes** ⬜ **No** ⬜ **Not reported** ⬜ **Not applicable**

Ethnicity: ⬜ **Yes** ⬜ **No** ⬜ **Not reported** ⬜ **Not applicable**

Social status: ⬜ **Yes** ⬜ **No** ⬜ **Not reported** ⬜ **Not applicable**

Disability: ⬜ **Yes** ⬜ **No** ⬜ **Not reported** ⬜ **Not applicable**

Literacy: ⬜ **Yes** ⬜ **No** ⬜ **Not reported** ⬜ **Not applicable**

Occupation: ⬜ **Yes** ⬜ **No** ⬜ **Not reported** ⬜ **Not applicable**

(incl vulnerable groups such as sex workers)

1. Is the language of the decision aid understandable/appropriate to young people of different:

Age ⬜ **Yes** ⬜ **No** ⬜ **Not reported** ⬜ **Not applicable**

Sex/Gender ⬜ **Yes** ⬜ **No** ⬜ **Not reported** ⬜ **Not applicable**

Ethnicity ⬜ **Yes** ⬜ **No** ⬜ **Not reported** ⬜ **Not applicable**

Social status ⬜ **Yes** ⬜ **No** ⬜ **Not reported** ⬜ **Not applicable**

Disability ⬜ **Yes** ⬜ **No** ⬜ **Not reported** ⬜ **Not applicable**

Literacy ⬜ **Yes** ⬜ **No** ⬜ **Not reported** ⬜ **Not applicable**

Occupation ⬜ **Yes** ⬜ **No** ⬜ **Not reported** ⬜ **Not applicable**

(incl vulnerable groups such as sex workers)

**What are the important considerations or aspects of this decision aid that make it equitable to young people?**

**What are the important considerations or aspects not met by this decision aid that make it inequitable to young people?**

**Considering the important aspects met and not met by this decision aid, what is the overall equitability of the decision aid for young people?**

**Instructions for determining overall suitability of the decision aid.**

The suitability of the decision aid is based whether important considerations and aspects are met or not met by the decision aid across the domains. A decision aid which met important considerations or aspects across domains is considered appropriate and suitable.

**What are the overall important considerations or aspects of this decision aid that make it suitable to young people?**

**What are the overall important considerations or aspects not met by this decision aid that make it unsuitable to young people?**

**Considering the important aspects met and not met by this decision aid, what is the overall suitability of the decision aid for young people?**

| Not Suitable | 🗙 Slightly suitable | Most suitable |
| --- | --- | --- |

**Key:**

Not suitable = decision aid has too many important aspects that are missing to be considered suitable for young people.

Slightly suitable = decision aid has some important aspects that are captured, but some important aspects are missing for the decision aid to be considered suitable for young people as it is.

Most suitable = decision aid has adequately captured most or all the important aspects of a decision aid that is considered suitable for young people.
