# Supplementary material for: Availability, acceptability and adoption of decision aids for HIV prevention and contraception for young people: a scoping review protocol
Source: BMJ Open. 2026 Mar 4;16(3):e106381. doi: 10.1136/bmjopen-2025-106381 (PMC12970088; doi:10.1136/bmjopen-2025-106381)
Supplement: Supplementary file 3 [file bmjopen-2025-106381.reviewer_comments.pdf]

PEER REVIEW HISTORY

BMJ Open publishes all reviews undertaken for accepted manuscripts. Reviewers are asked to complete a checklist review form and are provided with free text boxes to elaborate on their assessment. These free text comments are reproduced below.

ARTICLE DETAILS

Title (Provisional)

Availability, acceptability, and adoption of decision aids for HIV prevention and contraception for young people: A scoping review protocol

Authors

Kabonga, Itai; Kuguyo, Oppah; Mancitshana , Lindiwe; Chidhanguro, Kudzai; Munhenzva, Sharon; Masiyambiri , Fadzai; Ruhode, Nancy; Mangenah, Collin; Sibanda, Euphemia

---

VERSION 1 - REVIEW

**Reviewer**	**1**
**Name**	**Freij, Bishara J**
**Affiliation**	**Beaumont Children's Hospital, Pediatrics**
**Date**	**06-Nov-2025**
**COI**	**None**

Kabonga and colleagues plan on performing a scoping review evaluating the question of availability, acceptability, and adoption of decision aids for HIV prevention and contraception for young people 15-24 years of age. Only English language papers will be included in the review. The scope is global.

This is an ambitious project that I feel may produce results that will be difficult to interpret given the vast cultural, economic, and educational divide between locales where such aids are being used.

I have a few questions that the authors should address before fully implementing their study protocol:

(1) Under Stage 1 (page 5 of 27) - Identifying the research question - Will the investigators assess for the literacy levels of the decision aids? Will they assess the decision aids on whether or not they provide information on the local costs of drugs which can be a major factor on the use of prevention measures?

(2) Line 149: The authors plan to contact local organizations that conduct work on HIV prevention, .etc. Do they mean local organizations in Zimbabwe only? Since this is a paper

with a global aim, do they plan on identifying local organizations in other countries/continents? They should clarify their intentions and how they plan to accomplish that.

(3) Under Table 2, "General Information": More important than the year of publication is the time period during which a study was done. Presumably, developers of decision aids evaluate what had been previously done to assess efficacy and then refine and adapt tools for local use. Are newer decision aids more effective? If so, lumping the old with the new may be confusing and not reflective of where things stand at the present time. Such decision aids should be compared and perhaps analyzed in different time groupings.

(4) Table 2, "Implementation strategies or interventions": How will the authors assess whether a particular decision aid for HIV prevention or contraception had been validated before implementation at a given locale? Just because a decision aid exists does not mean it was appropriate to start with.

(5) Some decision aids are aimed at helping physicians or health care providers. Will these be excluded? The authors will collect "Context characteristics" (Table 2) but healthcare setting decision aids should be one specifically aimed at patients and not intended to help physicians make better recommendations.

(6) The 15-24-year age range is broad in terms of cognitive development. A 15-year-old and a 24-year-old may both be young (per WHO) but are not intellectually comparable. The understanding and effectiveness of decision aids is unlikely to be the same for the younger and older members of each cohort, which also adds limitations to data interpretation.

(7) Different decision aids will have variable content, design, language, and readability issues. How will the authors figure out what elements work and do not work as they attempt to inform the reader on the merits and limitations of various content items?

VERSION 1 - AUTHOR RESPONSE

**REVIEWER 1**	
Under Stage 1 (page 5 of 27) - Identifying the research question - Will the investigators assess for the literacy levels of the decision aids? Will they assess the decision aids on whether or not they provide information on the local costs of drugs which can be a major factor on the use of prevention measures?	Yes, we will determine whether the decision aid can be used by people of lower literacy.<br><br>Thank you. We agree with the comment on cost; and we have now included to the data extraction template whether the decision aids provide information on cost.
Line 149: The authors plan to contact local organizations that conduct work on HIV prevention, .etc. Do they mean local organizations in Zimbabwe only? Since this is a paper with a global aim, do they plan on identifying local organizations in other	Thank for highlighting this. We have now deleted this statement and will focus on the search strategies described above, as it will not be possible to contact organisations outside of Zimbabwe.

countries/continents? They should clarify their intentions and how they plan to accomplish that.	Please see **page 7, line 197-200 of tracked version.**
Under Table 2, "General Information": More important than the year of publication is the time period during which a study was done. Presumably, developers of decision aids evaluate what had been previously done to assess efficacy and then refine and adapt tools for local use. Are newer decision aids more effective? If so, lumping the old with the new may be confusing and not reflective of where things stand at the present time. Such decision aids should be compared and perhaps analyzed in different time groupings.	Thank you for this suggestion. We will include the period during which the study was done. Our narrative analysis will also take period into account as suggested by the reviewer.<br><br>We have included this statement in the data analysis paragraph;<br><br>We will also conduct narrative comparisons across time periods represented in the review.<br><br>Please see **page 9, line 269-270 of tracked version.**
Table 2, "Implementation strategies or interventions": How will the authors assess whether a particular decision aid for HIV prevention or contraception had been validated before implementation at a given locale? Just because a decision aid exists does not mean it was appropriate to start with.	Thank you for this. In the data abstraction process, will collect data on the development process of the decision aid, including any evaluation. Our suitability assessment tool (appendix 2) has the following questions to collect validation data of decision aids before implementation/use;<br>a) Was alpha testing of the decision aid done? If yes, among who?<br>b) Were the views of those that participated in alpha testing incorporated<br>c) Was beta testing of the decision aid done? If yes, among who?<br>d) Were the views of those that participated in beta testing incorporated?<br><br>Please see **Appendix 2, page 2, questions 8, 9, 10, and 11.**
Some decision aids are aimed at helping physicians or health care providers. Will these be excluded? The authors will collect "Context characteristics" (Table 2) but healthcare setting decision aids should be one specifically aimed at patients and not intended to help physicians make better recommendations.	We clarify that the review centres on decision aids for supporting user/patient decisions, rather than supporting correct decisions by health workers. Within the user/patient decision aids we will include all types of decision aids for HIV prevention and contraception - those that users (patients) can use independently, or those where health workers walk the user/patient through, or both. We have defined the various typologies of decision aids in the suitability assessment questionnaire (**Appendix 2) page 1, question 5**:

	Is the decision aid?<br><br>a) **patient centred/facing** *(can be used by the patient/user independently).*<br>*b)* **health worker centred/facing alone** *(cannot be used by the patient/user independently, health workers giving information to the client).*<br>*c)* **health worker centred/facing with client engagement** *(cannot be used by the patient independently. Health worker uses the decision aid together with client).*<br>**d) both** *(can be used by patients/users or health workers independently).*
The 15-24-year age range is broad in terms of cognitive development. A 15-year-old and a 24-year-old may both be young (per WHO) but are not intellectually comparable. The understanding and effectiveness of decision aids is unlikely to be the same for the younger and older members of each cohort, which also adds limitations to data interpretation.	Thank you for pointing this out. We agree with this and have added this sentence as a limitation:<br><br>Synthesis will include assessment of suitability of decision aids among young people aged 15-24 years, yet they are a heterogenous group whose characteristics may differ by setting and by age.<br><br>Please see **page 3, line82-84 of tracked version,**
Different decision aids will have variable content, design, language, and readability issues. How will the authors figure out what elements work and do not work as they attempt to inform the reader on the merits and limitations of various content items?	We will use the suitability assessment tool (**Appendix 2**) to assess the content, design, language and readability suitability for young people. In addition, given that acceptability and readability will likely differ by content, our narrative synthesis will include any reports from authors on feasibility, acceptability and useability of the decision aids.
